# Comparison of the Influence of Two Flap Designs on Periodontal Healing after Surgical Extraction of Impacted Third Molars

**DOI:** 10.5681/joddd.2011.001

**Published:** 2011-03-18

**Authors:** Seyed Ahmad Arta, Reza Pourabbas Kheyradin, Ali Hossein Mesgarzadeh, Bahador Hassanbaglu

**Affiliations:** ^1^ Assistant Professor, Department of Maxillofacial Surgery, Faculty of Dentistry, Tabriz University of Medical Sciences, Tabriz, Iran; ^2^ Professor Department of Periodontology, Faculty of Dentistry, Tabriz University of Medical Sciences, Tabriz, Iran; ^3^ Associate Professor, Department of Maxillofacial Surgery, Faculty of Dentistry, Tabriz University of Medical Sciences, Tabriz, Iran; ^4^ Dentist, Private Practice, Tabriz, Iran

**Keywords:** Impacted lower third molar, periodontal complications, Szmyd and 3-cornered flaps

## Abstract

**Background and aims:**

Impacted lower third molar is found in 90% of the general population. Impacted lower third molar surgery may result in periodontal complications on the distal surface of the adjacent second molar. The aim of this study was to evaluate the effect of flap design on the periodontal status of the second molar after lower third molar surgery.

**Materials and methods:**

Twenty patients, with an age range of 18-26 years, participated in the present study. The inclusion criteria consisted of the presence of bilateral symmetrical impacted third molars on panoramic radiographs. The subjects were randomly divided into two groups. The impactions on the left and right sides were operated by Szmyd and triangular flaps, respectively. Postoperative management and medications were similar for both groups. The subjects were evaluated at two-week, one-month, and six-month postoperative intervals by a surgeon who was blind to the results. Data was analyzed by t-test using SPSS 11 software.

**Results:**

There were no significant differences in clinical attachment loss, pocket depth, bone level, plaque index, and free gingival margin between the two flaps (p>0.05).

**Conclusion:**

The results of the present study did not show any differences in pocket depth, clinical attachment level, bone level and FGM (free gingival margin) between the two flap designs under study.

## Introduction

Mandibular third molars are found in 90% of the general population, with 33% of the people having at least one impacted molar.^1^ The high prevalence of impaction might be attributed to both genetic and environmental factors.^2^ Various reasons have been suggested for mandibular third molar surgeries.^3^ These causes generally include caries and their outcomes, germination disorders, periodontal problems, dentist’s diagnosis such as orthodontic problems, and cases with less infection potential like trauma, patient’s will and loss of the root.^3,4^ Extraction of impacted mandibular third molar often involves the incision of soft tissues and bone removal, with several side effects including edema, pain and periodical trismus; it also increases the risk of post-operative periodontal side effects.^5^ Therefore, formation of periodontal pockets, loss of clinical attachment, and loss of second molar bone are possible complications.^3,5^ As a result, the surgical procedure, especially the incision type used in the surgery of impacted teeth, seems to be crucial. Although the results reported by some investigators indicate no noticeable relation between the type of the flap and post-operative periodontal health different results have been reported in the short- and long-term.^5-7^ In addition, considering the fact that the exposure of the bone leads to bone loss even without osteoctomia^2^ and different types of flaps used, further studies are deemed necessary. Among different types of flaps, Szmyd flap, compared to the normal triangular flaps, would probably have much better effects, maintaining a stripe of tissue on the buccal surface of the second molar. According to the results of previous studies, flap type has no significant effect on the periodontal healing of the adjacent tooth. However, in some studies some differences in flap types have been observed regarding primary healing process of the wound and incidence rate of alveolar osteitis.^8,9^

Tooth impaction type has not been investigated sufficiently because it is a qualitative factor. In the present study, efforts were made to investigate the periodontal factors at various time intervals in matched sample environments in comparison to previous studies, by classification of Gregory and Pell for impacted teeth.

The aim of the present study was to compare the influence of two flap designs on periodontal healing after extraction of impacted third molars.

## Materials and Methods

The present clinical trial was performed in the Department of Oral and Maxillofacial Surgery, Faculty of Dentistry, Tabriz University of Medical Sciences, on patients who needed bilateral similar surgeries for impacted (split mouth) lower third molars and the results of surgery with two Szmyd and 3-cornered flaps were evaluated.

Twenty patients aged 18-26 were selected and the type of impaction and the need for surgery were determined and confirmed by radiography.

Inclusion criteria were impacted lower third molars, 18-26 years of age without any systemic diseases and the patients' consent to participate in the study. A customized acrylic stent was fabricated for reproducibility of measurements and identical direction of probing during follow-up sessions.

Periodontal probing depth was measured with a Williams-type probe. Local anesthesia was administered by 2% lidocaine to decrease pain because probing to determine bone level is painful.

Attachment level, probing depth, and bone level were measured twice.

All the surgeries were performed by one surgeon. All the patients who were candidates for surgery were randomly selected for the two above-mentioned flap designs on the right or left sides.

Epinephrine at a concentration of 1:80000 was used for local anesthesia.

Different methods of surgery were explained to all the patients who, then, signed consent forms.

Chlorhexidine mouthwash and 50-mg diclofenac sodium tablets were administered every 6 hours for 4 days to all the patients. No antibiotic prophylaxis was used. After 7 days, the sutures were removed.

Variables under study included patient gender, clinical attachment loss, pocket depth, bone level, plaque index and FGM (free gingival margin), which were evaluated before surgery (baseline) and at 2-week, one-month and six-month intervals after surgery.

Statistical analysis

Data was analyzed by SPSS software version 11.5 for Windows using paired-samples t-test and independent- samples t-test at a significance level of p<0.05.

## Results

Gingival indices were recorded at baseline in all the patients and considered as zero. Mean attachment loss values after Szmed flap surgery in male and female subjects were 7±1.99 and 6.79±1.69 mm, respectively.

There were no significant differences between male and female subjects in clinical attachment loss in group 1. The same results were achieved at 2- and 4-week intervals. The 6-month follow-up yielded the same results.

Comparison of the flaps did not reveal any differences between the two flap designs (p=0.621).

The two groups did not demonstrate significant differences in pocket depths at 2-week, 4-week, and 6-month intervals.

There were no significant differences in bone level between the two groups after two and four weeks and at 6-month follow-up. Table 1 summarizes the details.

Free gingival margin evaluation yielded the same results.

The results of the study are presented in Table 1.

**Table 1:** Comparison of clinical attachment loss, pocket depth, bone level, plaque index, and free gingival margin between the two flaps

Variable	3-cornered Flap	Szmyd Flap	P- value
Clinical attachment loss			
Baseline	6.80 ±1.20	6.55 ±1.66	0.452
Two week	7.10 ±1.28	6.95 ±1.69	0.609
One month	6.73 ±1.39	6.58 ±1.70	0.600
Six month	6.68 ±1.14	6.25 ±1.56	0.111
Pocket depth			
Baseline	1.65 ±.49	3 1.73 ±.5	0.591
Two week	1.65 ±.52	1.75 ±.47	0.447
One month	1.58 ±.41	1.65 ±.54	0.634
Six month	1.55 ±.43	1.50 ±.40	0.705
Bone level			
Baseline	9.95 ±1.18	9.98 ±.95	0.953
Two week	9.57 ±1.33	9.53 ±1.06	0.914
One month	9.38 ±1.32	9.35 ±1.11	0.959
Six month	9.18 ±1.35	9.20 ±1.03	0.958
Plaque index			
Baseline	10.00 ±1.72	10.00 ±1.92	0.572
Two week	9.55 ±1.88	10.15 ±2.16	0.083
One month	9.40 ±2.09	9.70 ±2.05	0.209
Six month	9.10 ±1.68	9.20 ±1.94	0.649
Free gingival margin			
Baseline	5.15 ±1.03	4.83 ±1.48	0.295
Two week	5.40 ±1.15	5.20 ±1.53	0.519
One month	5.15 ±1.18	4.93 ±1.52	0.353
Six month	5.13 ±1.12	4.75 ±1.42	0.167

Gingival index between the two groups was zero in the present study.

## Discussion

Surgery methods, especially type of flap incision, have an important role in the periodontal health of the mandibular second molar after extraction of the adjacent impacted third molar.

Results of several studies have shown that flap design has no correlation with periodontal health status of the mandibular second molar after the extraction of the adjacent impacted third molar but different short and long-term results of these correlations have been reported.,^6^

More radiographic bone loss was found at the sites adjacent to the surgical location than at the sites distant to the surgical location.^2^

Of several types of flap, Szmyd flap had better periodontal healing of second molars after fully-impacted mandibular third molar extractions.

Kirtiloğlu et al^9^ demonstrated that the mean probing depth (PD) at distal and buccal sites was significantly different between the flaps at 1-week, 2-week, and 4-week intervals postoperatively (p<0.05). There were no significant differences in preoperative and 1-year postoperative mean PD between the two flap designs (p>0.05). There were no significant differences in mean clinical attachment levels between the flap sites after one year (p>0.05). In addition, the modified Szmyd flap, which leaves intact gingiva around the second molar, has better primary periodontal healing than the 3-cornered flap after surgical removal of the fully-impacted mandibular third molar.^9^

Rosa et al did not demonstrate any statistically significant differences in measurements of probing depth, clinical attachment level, or bone level for the two types of flaps used or the two surfaces measured.^10^

In our study no significant differences were observed in pocket depths, clinical attachment levels, bone levels and FGMs (free gingival margin) between the two flap designs (p>0.05).

Jakse et al,^11^ in evaluation of the two different flap designs, demonstrated that the Szmyd flap in lower third molar surgery considerably influences primary wound healing. The modified triangular flap is significantly less conducive to the development of wound dehiscence. In another study, de Brabander et al^12^ removed molars using a mucoperiosteal flap as described by Szmyd and analysis of variance indicated that there was no significant difference between the two types of wound closure.

Karaca et al^13^ demonstrated that selection of a flap design does not seem to have a lasting effect on the health of periodontal tissue. Results of the present study and several other studies demonstrate no significant differences in pocket depth, clinical attachment level, bone level and FGM between the two flap designs.

## Conclusion

No significant differences were observed between baseline, two-week, one-month and three-month intervals in pocket depth, bone level, plaque index, and FGM between Szmyd and 3-cornered flap (p>0.05). The results of the present study and several other studies demonstrate no significant differences between pocket depth, clinical attachment level, bone level and FGM between the two flap designs.
